# Clinical Implications of Color Adjustment in Single-Shade Resins Post-Dental Bleaching: A Systematic Review

**DOI:** 10.3390/jcm14093194

**Published:** 2025-05-05

**Authors:** Samille Biasi Miranda, Caroline de Farias Charamba Leal, Rodrigo Barros Esteves Lins, Marcos Antonio Japiassu Resende Montes

**Affiliations:** 1 Departament of Dental Materials, Faculty of Dentistry, University of Pernambuco, Recife 50100-130, PE, Brazilmarcos.japiassu@upe.br (M.A.J.R.M.); 2 Departament of Restorative Dentistry, School of Dentistry, Federal University of Alagoas, Maceió 57072-900, AL, Brazil; rodrigo.lins@foufal.ufal.br

**Keywords:** composite resins, tooth bleaching, color, systematic review

## Abstract

**Background**: Tooth bleaching can compromise the color match between dental tissues and restorations. Single-shade resin-based composites (RBCs) have been developed to simplify shade matching; however, their performance after dental bleaching remains uncertain. **Objectives**: This study aimed to evaluate the ability of single-shade RBCs to adapt to the color of the dental substrate after dental bleaching. **Methods**: A systematic review was conducted according to the PRISMA guidelines and registered in PROSPERO. Searches were performed in PubMed, Embase, Web of Science, and Scopus (December 2024). In vitro studies were selected based on the PICOS criteria (P: human or bovine teeth; I: teeth restored with single-shade RBCs and bleached; C: bleached dental tissue; O: color match; S: in vitro studies). Two reviewers (kappa = 0.90) applied eligibility criteria, extracted the data, and assessed the risk of bias using the RoBDEMAT tool. **Results**: Eight studies involving 362 restorations were included. Most studies indicated that single-shade RBCs achieved an acceptable color match after tooth bleaching. Study quality was generally moderate to low, with most evaluations rated as “sufficiently reported or adequate”. **Conclusions**: Single-shade RBCs demonstrated the ability to match bleached dental tissue in vitro, although effectiveness may vary depending on bleaching duration and storage conditions.

## 1. Introduction

The aesthetics of a smile are influenced by intrinsic dental factors such as tooth shape, size, proportion, axial inclination, color, and contact areas [1]. Among these, tooth color plays a crucial role in creating a harmonious smile, often being the first feature patients notice [2]. Dissatisfaction with tooth color, especially in individuals who have not undergone cosmetic treatments, can lead to psychosocial challenges [3]. Among the available options for enhancing tooth color, dental bleaching stands out due to its simplicity, effectiveness, and cost-efficiency [4].

Dental bleaching techniques can be classified based on the setting (in-office or at-home) and the type of dental element involved (vital or non-vital) [5]. According to the “chromophore theory”, bleaching agents oxidize tooth pigments, producing oxygen and superoxide radicals that penetrate the dental structure, breaking down larger pigments into smaller, less visible molecules [4]. Nevertheless, the bleaching process poses challenges in the presence of existing restorations, as these materials may not respond predictably to bleaching procedures [6], and although composite resins may exhibit some degree of color change following bleaching, studies have indicated that such changes are often not clinically significant [7].

Notably, composite resins generally do not lighten to match bleached teeth, leading to a mismatch between the restoration and the surrounding bleached dental structure [8]. This discrepancy often leads to patient dissatisfaction and the need to replace restorations after bleaching, resulting in additional costs and the potential loss of healthy tooth structure [8,9].

Recently, single-shade resin-based composites (RBCs) were introduced, utilizing “smart monochromatic technology” to replicate the color of adjacent teeth, potentially simplifying restorative procedures by eliminating the need for multiple shades [10,11]. Some studies suggest that, while bleaching reduces post-pigmentation color change in these materials, their original shade is not fully restored [12]. The color-matching potential of single-shade RBCs appears to be more effective in lighter shades compared to darker ones [10].

Therefore, it is necessary to evaluate whether single-shade RBCs can match the color of bleached teeth, especially given the increasing demand for esthetic restorations that blend seamlessly with bleached dental substrates. Despite its clinical efficacy, no systematic review has yet synthesized the available evidence on this topic. Therefore, the objective of this study was to investigate the ability of single-shade resins to adapt their coloration to bleached dental substrates, providing a scientific basis to guide clinical practice and improve patient outcomes. The null hypothesis to be tested is that the use of single-shade resin composites does not achieve a clinically acceptable color match in teeth following dental bleaching.

## 2. Materials and Methods

### 2.1. Protocol and Registration

This systematic review followed the Preferred Reporting Items for Systematic Reviews and Meta-Analyses (PRISMA) guidelines (Supplementary Material Table S1) [13]. The study protocol was registered in the International Prospective Register of Systematic Reviews (PROSPERO) under the registration number #CRD42024539437.

### 2.2. Eligibility Criteria

The review was guided by the research question: “Are single-shade resin-based composites (RBCs) able to adapt to the color of the dental substrate after dental bleaching?”. Using the PICOS strategy, the population (P) consisted of extracted human or bovine teeth, the intervention (I) involved restoration with single-shade resin-based composites (RBCs) followed by a bleaching procedure, the comparator (C) was bleached dental tissue, the outcome (O) assessed was the color match of the restorations, and the study design (S) included in vitro studies.

The inclusion criteria were in vitro studies evaluating the color match of single-shade RBCs after dental bleaching (either at-home or in-office), studies conducted on sound human teeth, and studies that included a control group. The exclusion criteria were studies involving teeth with caries or enamel defects, studies on devitalized teeth, and studies involving over-the-counter products.

### 2.3. Information Sources and Search Strategy

A search was conducted in PubMed, Embase, Web of Science, and Scopus databases in May 2024, and updated in December 2024. No chronological limits were applied. The search terms used included Medical Subject Headings (MeSH) and text words: [(“Single-shade composite” OR “monoshade universal composite” OR “monochromatic composite resin” OR “resin AND chameleon AND effect”) AND (“Color match” OR “color matching” OR “color stability” OR “color adjustment” OR “color blending” OR “color adapting” OR “chromatic stability” OR “color correction”) AND (“Tooth Bleaching” OR “teeth whitening” OR “teeth bleaching” OR “dental bleaching” OR “whitening” OR “bleaching”)] (Table 1).

### 2.4. Selection Process

Study selection was managed using Rayyan (Qatar Computing Research Institute). After duplicate removal, titles and abstracts were screened to verify they met the predefined criteria. The selection process was conducted independently by two reviewers (S.B.M. and C.d.F.C.L.), who were previously calibrated. Discrepancies were resolved through discussion with a third reviewer (R.B.E.L.). Calibration involved reviewing 10 articles together to align the reviewers’ interpretation of the criteria (kappa 0.90). Eligible studies then underwent a full-text review for data extraction.

### 2.5. Data Collection Process

Data extraction was independently performed by two authors (S.B.M. and C.d.F.C.L.), using a standardized table to register the following fields: author/year, study aim, restorative materials, sample size, bleaching type (in-office or at-home), bleaching agent, analysis tools, color measurement background, follow-up duration, storage media, and the main results/conclusion.

### 2.6. Study Risk of Bias Assessment

The risk of bias in included studies was assessed using the RoBDEMAT tool [14], designed for in vitro dental materials research. Bias was assessed across four domains: D1 related to planning and allocation, including the use of a control group, randomization, and sample size determination; D2 concerned specimen preparation, with emphasis on standardization of samples and materials and maintaining identical experimental conditions; D3 addressed outcome assessment through standardized testing, defined outcome measures, and operator blinding; and D4 focused on data treatment and outcome reporting, including appropriate statistical analysis and complete reporting of outcomes.

Each item was classified as “sufficiently reported/adequate”, “insufficiently reported”, “not reported/not adequate”, or “not applicable”. Any disagreements between assessors were resolved by consulting a third reviewer (R.B.E.L.).

### 2.7. Effect Measures and Synthesis Methods

The heterogeneity among the included studies was evaluated using Cochran’s Q test and the I^2^ index. The Q value was 556.17, and the I^2^ index was 98.38%, indicating high heterogeneity.

## 3. Results

### 3.1. Study Selection

**Figure 1:**
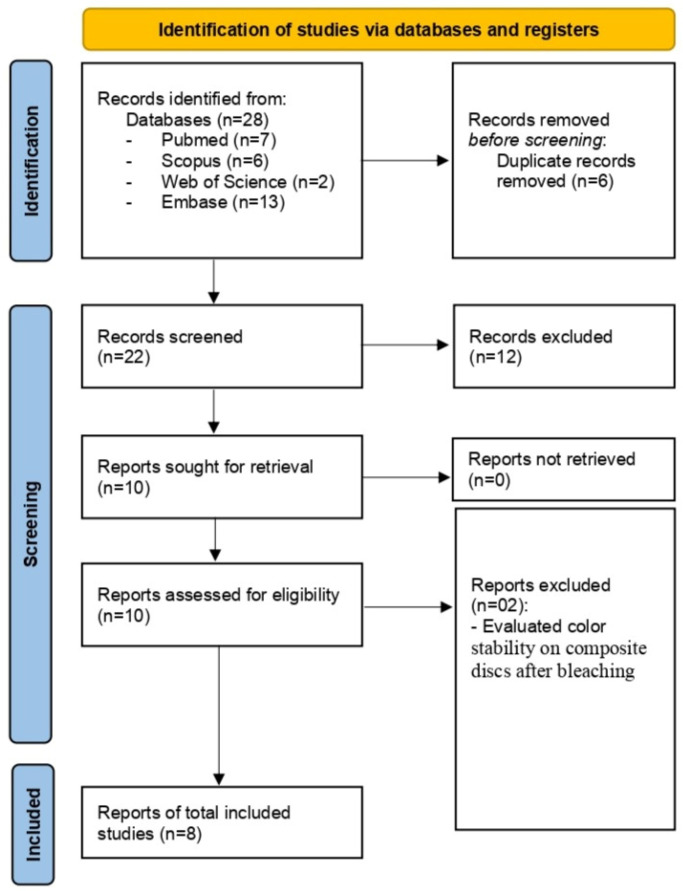
PRISMA flow diagram of the literature search and selection criteria.

A total of 28 studies were initially identified from the database search conducted in August 2024 (Updated in December 2024). After removing duplicates, 22 studies remained, and their titles and abstracts were screened based on the predefined inclusion and exclusion criteria. Ten studies were considered potentially eligible and underwent full-text review. Of these, eight met the selection criteria (Figure 1). Two studies were excluded because they assessed color stability on composite discs, rather than evaluating color adjustment in bleached teeth [12,15].

### 3.2. Study Characteristics

The compositions of the single-shade RBCs evaluated in the studies included in this systematic review are listed in Table 2.

The studies were published between 2020 and 2024 and evaluated a variety of single-shade RBCs, including Omnichroma (Tokuyama Dental, Tokyo, Japan), Venus Diamond One (Kulzer GmbH, Hanau, Germany), Essentia Universal (GC Corporation, Tokyo, Japan), A’Uno Universal Basic (Micerium S.p.A., Avegno, Italy), Transcend Universal Composite (Ultradent Products, South Jordan, UT, USA), and Beautifil Unishade (Shofu Inc., Kyoto, Japan). The comparator RBCs used were Clearfill Majesty Esthetic and ES-2 Universal. Sample sizes ranged from 6 to 80 permanent teeth, with one study using bovine incisors [16]. The studies primarily involved posterior teeth restored with Class V cavities [17,18,19,20,21,22], incisors with cavities measuring 3 × 5 mm in width and 2 mm in depth [23], and bovine incisors with cavities of 3 × 2 mm [16]. Follow-up periods varied, ranging from baseline to 28 days post-bleaching [16,17,18,19,20,21,22]. In total, 362 teeth were restored across the eight studies [16,17,18,19,20,21,22]. These characteristics of the included studies are summarized in Table 3 and Table 4.

Among the included studies, seven utilized in-office bleaching [16,17,18,19,20,21,22,23], and one study involved at-home bleaching [23]. Regarding the in-office bleaching, six studies used Opalescence Boost 40% (Ultradent Products, South Jordan, UT, USA) [17,18,19,20,22,23], one used Opalescence Boost 35% (Ultradent Products, South Jordan, UT, USA) [16], and one used Power Whitening Gel 40% (FGM Dental Group, Joinville, SC, Brazil) [21]. The at-home bleaching study used Opalescence 16% (Ultradent Products, South Jordan, UT, USA) [23]. Color assessment was performed using different instruments: five studies used Vita Easyshade V (VITA Zahnfabrik, Bad Säckingen, Germany) [17,18,19,20,21], one used SpectroShade™ Micro (MHT Optic Research AG, Niederhasli, Switzerland) [23], one used Crystaleye Spectrophotometer (Olympus Corporation, Tokyo, Japan) [22], and one used CMS-35F SQC (Murakami Color Research Laboratory, Tokyo, Japan) [16].

Color measurements were taken from several locations: the center of the Class V restoration [17], 1 mm from the restoration margin [18,19,20], 2 mm from the margin, including the incisal and cervical regions [16], across the surrounding teeth in three sections, and directly over the filling [23], as well as from both the restoration color and the adjacent natural tooth structure [21]. Background colors varied between studies, including grey [18,20], white [16,21,23], and black [16,22], while two studies did not specify the background color [17,19].

Almost all studies used distilled water as the storage medium, except for one study that used coffee [23] and another that used black tea [16]. Color match outcomes were reported using the L*, a*, and b* coordinates, color variations according to CIEDE 2000 (ΔE00), CIELAB (ΔEab), and changes in the L* value. One study mentioned using the Easyshade device for color match analysis but only reported visual color assessment data. The impact of at-home versus in-office bleaching on the color match of RBCs was assessed in one study [23], which found that in-office bleaching had a lesser effect on the color match between the tooth and the restoration.

Five studies [16,17,18,21,22] did not use a multishade RBC for comparison with single-shade RBCs. Alhabdam et al. [17] reported that single-shade RBCs showed a different color match compared to adjacent teeth, with the ability to appear lighter after coffee exposure and bleaching. Razzaq, Refaat, and Al-Badr [21], along with Forabosco and Cecchi [18], observed that single-shade RBCs could adapt to tooth shade post-bleaching. Sugimura et al. [16] found that after the second bleaching application, single-shade RBCs struggled to maintain a color match with the enamel. Mohamed et al. [22] reported that single-shade RBCs changed to a lighter shade in response to bleaching, especially when the enamel color changed, but showed no change when the enamel remained stable. However, color stability was not maintained two weeks after bleaching [17].

When comparing single-shade and multishade RBCs, Cubukcu et al. [23] found that single-shade RBCs exhibited a weaker color match with the tooth. In contrast, Forabosco et al. [19] and Forabosco et al. [20] reported that single-shade RBCs showed a better color match after bleaching.

### 3.3. Risk of Bias in Studies

The risk of bias assessment is presented in Table 5. While none of the included studies were rated as “sufficiently reported/adequate” across all RoBDEMAT items, the most common judgment overall was “sufficiently reported/adequate”, especially in D1: Bias in planning and allocation, where all studies included a control group and most ensured identical experimental conditions. In D4: Bias in data treatment and outcome reporting, most studies used appropriate statistical analysis and clearly reported their outcomes. However, common weaknesses were found in sample size calculation and randomization (D1), standardization of testing procedures (D2), and blinding of the test operator (D3), suggesting an overall moderate to low methodological quality.

### 3.4. Summary Measures and Synthesis Methods

Methodological differences were observed, particularly regarding follow-up periods (e.g., baseline, immediately post-bleaching, 24 h, 1 week, 2 weeks, 4 weeks) and storage media (coffee, distilled water). Given this heterogeneity, a meta-analysis was deemed inappropriate. Instead, a detailed qualitative synthesis was conducted, focusing on the outcomes and data extracted from the included studies.

## 4. Discussion

**Figure 2:**
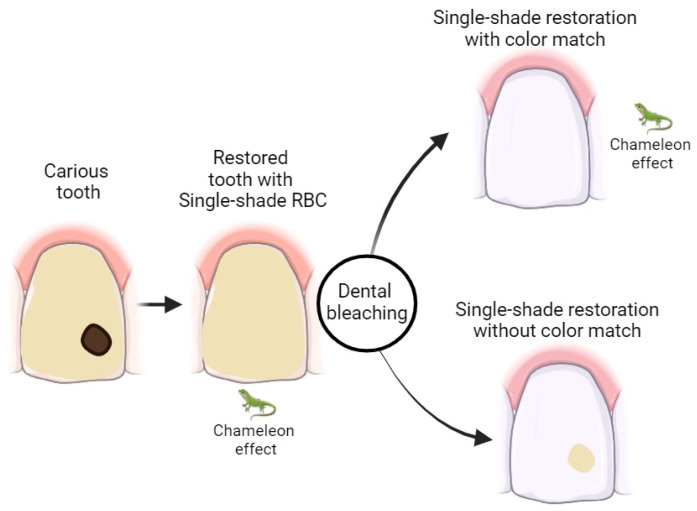
Restored tooth after dental bleaching.

This systematic review aimed to examine whether single-shade RBCs can effectively adjust their color to match bleached teeth (Figure 2). The null hypothesis—that the use of single-shade resin composites does not achieve a clinically acceptable color match following dental bleaching—was rejected. The majority of the included in vitro studies demonstrated that single-shade RBCs possess the potential to achieve an acceptable color adaptation to bleached teeth, although results varied depending on factors such as bleaching protocols, evaluation times, and storage conditions.

More translucent composite resins can reflect the color of the dental substrate they are applied to, resulting in a color change phenomenon known as the “chameleon effect” [24]. Single-shade RBCs were introduced to the market with the promise of mimicking tooth color using only one shade, thanks to the presence of “smart monochromatic technology” [11]. A previous systematic review that compiled clinical trials assessing the color stability of single-shade resins found that these innovative resins exhibited similar performance in both color match and color stability compared to multi-shade resins on non-bleached dental substrates [25]. However, this review specifically focused on the behavior of these materials following dental bleaching—an area previously underexplored.

The included studies generally supported the ability of single-shade RBCs to achieve good color matching after bleaching. The CIE Lab system characterizes color using three coordinates: L* (lightness), a* (chroma in the red–green axis), and b* (chroma in the yellow–blue axis) [17]. Mohamed et al. [22], in a study on class V cavity-restored teeth, found that the L* values of the restoration enamel remained consistent across all evaluation periods post-bleaching, indicating a good color match. The L*, a*, and b* values of the restorations closely matched the surrounding enamel, supporting the idea of color adaptation of Omnichroma resin in class V restorations [22]. Similarly, Razzaq et al. [21] found no statistical difference in the L* coordinate between the tooth and the restoration, suggesting good color correspondence after bleaching in teeth with class V restorations.

In contrast, Alhabdan et al. [17] found statistical differences in the L* and b* coordinates between Omnichroma resin and bleached dental structures. Positive ΔL values indicated a shift toward a lighter shade, implying that Omnichroma resin appeared lighter and thus did not achieve a good color match in class V restorations [16]. These inconsistencies may be attributed to a lack of standardization in tooth color across the samples. A previous study [16] noted that matching ability is influenced by the initial tooth color evaluated. Sugimura et al. [15] observed a trend of increasing ΔL values with more bleaching sessions. However, their results were based on worn bovine enamel specimens, which differ from human enamel in translucency and surface characteristics, potentially affecting interpretation. Thus, differences between studies should be interpreted with caution, as there was heterogeneity among experiments, particularly regarding evaluation time post-bleaching, storage media, and sample size.

The L*, a*, and b* coordinates are used in formulas to calculate ΔE values using CIELab, resulting in ΔEab, and using CIEDE2000, resulting in ΔE00 [17]. It is reported that CIEDE2000 is considered more representative of human visual perception [26]. An ΔE value of 0 indicates no color change between the compared samples. ΔE values between 0 and 3.2 represent a color change that is not visually perceptible and is typically clinically acceptable. However, ΔE values of 3.3 or higher indicate a visually noticeable color change that may be considered clinically unacceptable [26]. Three studies reported their results using ΔE00 [17,19,23], while three used ΔEab [16,20,21].

Razzaq et al. [21], observed a reduction in ΔEab after dental bleaching between the dental structure and the restoration, indicating improved color correspondence. Furthermore, no statistical difference was detected in ΔEab between the dental structure and the restoration, further confirming a good color match between them following dental bleaching. Forabosco et al. [19,20] similarly found that single-shade RBCs like Omnichroma, Venus Diamond One, and Essentia Universal achieved excellent color matching after dental bleaching. Omnichroma single-shade RBCs showed superior color correspondence to post-bleaching shades on the VITA scale [19]. Forabosco and Cecchi [18], in a pilot study, also reported excellent pre- and post-bleaching color matches with single-shade RBCs Omnichroma and Venus Diamond One.

In contrast, Sugimura et al. [16] found that none of the evaluated resins achieved acceptable color matches when ΔEab and ΔE00 values were interpreted according to the 50:50% perceptibility and acceptability thresholds. The ΔE00 values found by Cubukcu et al. [23] also reported ΔE00 values below the clinically acceptable threshold, suggesting poor color adaptation. Their study involved coffee immersion over five days to simulate five years of staining and found that at-home bleaching produced poorer matches than in-office bleaching [23]. Alhamdan et al. [27] emphasized the influence of the storage media on results, while Alhabdan et al. [17], who used distilled water for storage, reported ΔE00 values exceeding the perceptible threshold of 3.3—implying visually noticeable differences, with resins appearing lighter than the surrounding enamel [17].

The ΔE00 and ΔEab values were compared based on the 50:50% perceptibility and acceptability thresholds. The 50:50% acceptability threshold for color difference (AT) represents the level at which half of the observers find the color variation acceptable under controlled conditions, while the other half would recommend replacing or adjusting the restoration [28]. According to ISO/TR 28642:2016, ΔEab values of 1.2 and 2.7, and ΔE00 values of 0.8 and 1.8 are the defined perceptibility and acceptability thresholds, respectively. Similarly, ΔE00-CIEDE 2000 values were analyzed according to the same perceptibility and acceptability standards, with thresholds of 0.8 and 1.8, respectively, as established by previous studies [28].

Most studies reported ΔE00 values above the 1.8 acceptability threshold, indicating that the perceptible color difference is significant, especially shortly after bleaching. For instance, the study by Cubukcu et al. [23] reported elevated ΔE00 values (14.49) one day after bleaching for single-shade resin restorations with in-office bleaching, which decreased to values closer to acceptability over time. Forabosco et al. [18] also observed a progressive decrease in ΔE00 values across different restoration techniques, with some variations remaining above the acceptability threshold even after one week. These findings suggest that, while materials may approach the original tooth color over time, the initial change is generally noticeable and often clinically unacceptable.

Regarding ΔEab values, the results reflect similar trends to those of ΔE00, with most values exceeding the acceptability limit (ΔEab = 2.7). Notably, the studies by Forabosco et al. [20] report high ΔEab values, particularly at baseline (OM with ΔEab = 12.5 and CL with ΔEab = 12.6), followed by a significant reduction after 24 h and one week. Razzaq et al. [21] and Sugimura et al. [16] demonstrate that restorative resins exhibit noticeable color variations after multiple applications, with ΔEab values increasing as more layers of material are applied. Although reductions are observed over time, the fact that many values remain above the acceptability threshold suggests that these restorations may still not achieve ideal color matching, even after the initial curing period, underscoring the need for improved materials for better post-bleaching stability.

The color matching potential of single-shade composite resins relies on the optical interaction with dental tissues and the substrate translucency, allowing for the so-called “chameleon effect” [29]. However, after dental bleaching with 35% hydrogen peroxide, studies show that enamel may become slightly more opaque due to superficial demineralization and increased roughness, which reduces translucency and light transmission [30]. This analysis was conducted one week after bleaching, indicating that the observed changes in translucency reflect a recent phase, prior to the complete optical stabilization of the dental structure. Although subtle, this alteration may negatively impact the ability of single-shade resins to integrate chromatically with the bleached tooth, especially in areas where enamel is the predominant tissue. Thus, the reduction in translucency following bleaching may limit the aesthetic performance of these resins, requiring careful consideration when selecting the restorative material. Moreover, the color match of single-shade composites appears to be both time- and media-dependent, with factors such as aging, hydration, light conditions, and chemical environment modulating the appearance and adaptation of restorative materials over time [11,17,18].

The color matching of single-shade resins is influenced by cavity wall thickness, with thinner walls, especially those of 1 mm, resulting in greater color differences (ΔE00) and lower adjustment capabilities [28]. These findings underscore the importance of considering not only the material properties but also the structural factors of the dental element. Additionally, the dental substrate color can affect the color match of single-shade resins, with less color discrepancy observed on A1 substrates compared to A3 [31]. It is essential for future studies to specify the dental element colors in their samples, as this can impact the results. Among the reviewed studies, only two mentioned this information [22,23]. The included studies used different background colors such as black [22], grey [18], and white [17], which could influence the evaluation of color adjustment capacity. More translucent composites used on bleached substrates are prone to greater color shifts depending on the background color [32].

There was heterogeneity among the included studies regarding evaluation methods and bleaching protocols. Studies that included multiple bleaching protocols demonstrated that home bleaching induced less perceptible color change than in-office bleaching [22,23]. Additionally, there was inconsistency regarding the duration of specimen storage post-bleaching. While most studies performed evaluations after 24 h of storage [21,22], others extended the evaluation period up to 28 days [17,19]. One study standardized the storage period across different methodologies by evaluating samples at various time points post-bleaching [22]. Furthermore, there is a lack of consensus on the type of control groups used. For example, some studies used restorations with multiple shades as a control group [22], while others evaluated restorations with different types of bleaching treatments without a multi-shade comparator [17]. The lack of standardized control groups limits the comparability of the included studies. Future studies should address this by employing consistent control groups and bleaching protocols to enhance the robustness of the findings.

This review identifies several challenges in the studies analyzed. Firstly, there is a considerable variation in study design, including differences in follow-up periods after bleaching, storage methods, and approaches to color assessment. This diversity complicates direct comparisons across studies. Secondly, the quality of the included studies was generally rated as moderate to low based on the RoBDEMAT tool, revealing issues such as inadequate reporting of randomization, lack of standardized procedures, and insufficient use of blinding techniques. These methodological flaws undermine the internal validity of the studies. Thirdly, the use of small sample sizes and extracted teeth in laboratory settings limits the applicability of the findings to clinical scenarios, especially when considering diverse patient populations. Additionally, due to the heterogeneity among studies, a detailed quantitative meta-analysis was not feasible, which may affect the strength of the conclusions drawn from this review. Most studies also had relatively short follow-up periods, which might not adequately reflect the long-term color stability and durability of single-shade resins.

Lastly, while laboratory conditions provide controlled environments for evaluation, real-life factors such as patient behavior, oral hygiene practices, and natural wear over time can significantly impact the clinical relevance of these findings. Acknowledging these limitations is essential for the accurate interpretation of results and for guiding future research. Researchers are encouraged to meticulously document key details in future in vitro studies, including blinding of outcome assessors, storage conditions, methods for sample randomization and standardization. Additionally, following established methodologies from previous studies can enhance comparability and allow for more robust meta-analyses in the future. Furthermore, it is important to highlight that, this review not only identifies the potential and current limitations of single-shade RBCs but also emphasizes the necessity for rigorous methodological standards in future studies. Improved standardization will help ensure more reliable results and strengthen the clinical relevance of these materials, ultimately contributing to better patient outcomes.

## 5. Conclusions

This systematic review demonstrated that single-shade RBCs offer promising color adaptation capabilities after dental bleaching, with several studies reporting good color matches with surrounding dental tissues. However, the significant heterogeneity in the methodologies used across the studies raises concerns about the consistency and reliability of the findings. While some data suggest that single-shade RBCs may not always achieve clinically acceptable ΔE levels, the variability in evaluation criteria, bleaching protocols, and color assessment methods underscores the need for more standardized approaches in future research.

## Figures and Tables

**Table 1:** Search strategy.

Database	Search Strategy
Pubmed	(“Single-shade composite*” [All Fields] OR (“monoshade*” [All Fields] AND “universal*” [All Fields] AND “composite*” [All Fields]) OR (“monochromatic*” [All Fields] AND “composite*” [All Fields] AND “resin*” [All Fields]) OR (“resin*” [All Fields] AND “chameleon effect*” [All Fields])) AND (“Color match” [All Fields] OR “color matching” [All Fields] OR “color stability” [All Fields] OR “color adjustment” [All Fields] OR “color blending” [All Fields] OR (“color” [All Fields] AND “adapting” [All Fields]) OR “chromatic stability” [All Fields] OR “color correction” [All Fields]) AND (“Tooth Bleaching” [All Fields] OR “teeth whitening” [All Fields] OR “teeth bleaching” [All Fields] OR “tooth bleaching” [All Fields] OR “dental whitening” [All Fields] OR “dental bleaching” [All Fields] OR “bleaching” [All Fields] OR “whitening” [All Fields])
Embase	(‘Single-shade composite’ OR ‘Single-shade composites’ OR (‘monoshade’ AND ‘universal’ AND ‘composite’) OR (‘monochromatic’ AND ‘composite’ AND ‘resin’) OR (‘resin’ AND ‘chameleon effect’)) AND (‘Color match’ OR ‘color matching’ OR ‘color stability’ OR ‘color adjustment’ OR ‘color blending’ OR ‘color adapting’ OR ‘chromatic stability’ OR ‘color correction’) AND (‘Tooth Bleaching’/exp OR ‘teeth whitening’/exp OR ‘teeth bleaching’/exp OR ‘tooth bleaching’/exp OR (‘dental AND whitening’) OR (‘dental AND bleaching’) OR ‘bleaching’ OR ‘whitening’)
Web of Science	TS = (“Single-shade composite” OR “monoshade universal composite” OR “monochromatic composite resin” OR “resin chameleon effect”) AND TS = (“Color match” OR “color matching” OR “color stability” OR “color adjustment” OR “color blending” OR “color adapting” OR “chromatic stability” OR “color correction”) AND TS = (“Tooth Bleaching” OR “teeth whitening” OR “teeth bleaching” OR “tooth bleaching” OR “dental whitening” OR “dental bleaching” OR “bleaching” OR “whitening”)
The Cochrane Library	(Single-shade composite) OR (monoshade universal composite) OR (monochromatic composite resin) OR (resin chameleon effect) in Title Abstract Keyword AND (Color match) OR (color matching) OR (color stability) OR (color adjustment) OR (color blending) OR (color adapting) OR (chromatic stability) OR (color correction) in Title Abstract Keyword AND (Tooth Bleaching) OR (teeth whitening) OR (teeth bleaching) OR (tooth bleaching) OR (dental whitening) OR (dental bleaching) OR (bleaching) OR (whitening) in Title Abstract Keyword—(Word variations have been searched)
Scopus	TITLE-ABS-KEY(“Single-shade composite” OR “monoshade universal composite” OR “monochromatic composite resin” OR “resin chameleon effect”) AND TITLE-ABS-KEY(“Color match” OR “color matching” OR “color stability” OR “color adjustment” OR “color blending” OR “color adapting” OR “chromatic stability” OR “color correction”) AND TITLE-ABS-KEY(“Tooth Bleaching” OR “teeth whitening” OR “teeth bleaching” OR “tooth bleaching” OR “dental whitening” OR “dental bleaching” OR “bleaching” OR “whitening”)

**Table 2:** Material specifications and compositions of single-shade RBCs.

Single-Shade RBC	Manufacturer	Composition
Venus Diamond One	Kulzer GmbH (Hanau, Germany)	Matrix: UDMA, TEGDMA, TCD-DI-HEA. Filler system: BaAlF, SiO_2_ (64 vol.-%; 81 wt%; 5 nm–20 μm in ø)
Omnichroma	Tokuyama Dental, Tokyo, Japan	Matrix: TEGDMA, UDMA, Dibutyl hydroxyl toluene and UV absorber, Mequinol. Filler system: SiO_2_, ZrO_2_ (68 vol.-%; 79 wt%; 0.2–0.4 μm)
Essentia Universal	GC Corp. (Tokyo, Japan)	Matrix: UDMA. Filler system: Sr, LaF_3_, SiO_2_, FAISi glass, Fumed silica (81 wt.%; 16 μm–16 nm)
A Uno Universal Basic	Yamakin (Konan, Japan)	Resin composite: UDMA, bis-GMA, TEGDMA, Fluoride Sustained Release Glass Filler, aggregated SiO_2_-Al2O_3_-ZrO_2_ cluster, SiO_2_, others
Beautifil Unishade	Shofu (Kyoto, Japan)	Glass powder, Bis-GMA, Bis-MEPP, UDMA, TEGDMA
Transcend Universal Composite	Ultradent Products	dimethacrylate resin, methacrylate resin, silica, silane treated silica

Legend: AUDMA = Aromatic Urethane Dimethacrylate; TEGDMA = Tetraethylene glycol dimethacrylate; UDMA = Urethane Dimethacrylate; TCD-DI-HEA = Triethylene Glycol Dimethacrylate Diacrylate.

**Table 3:** Data extracted.

Study	RBC	Sample	Type of Bleaching	Analysis Tool	Color Background	Follow-Up	Storage Media	Conclusion
Mohamed et al. (2020) [22]	OM	Class V (A1-D4 shade)	In-office (Opalescence Boost 40%)	Crystaleye Spectrophotometer	Black	Baseline, 24 h, 1 week, 2 weeks and 4 weeks post-bleaching	Distilled water at 37 °C	OM lightened with enamel responsive to bleaching and remained unchanged with enamel unresponsive to bleaching.
AlHabdan et al. (2022) [17]	OM	Class V	In-office (Opalescence Boost 40%)	VITA Easyshade V	*	Immediately after bleaching and 2 weeks post-bleaching	Distilled water at 37 °C	After bleaching, OM lightened with surrounding enamel, but the color change was not stable after 2 weeks.
Forabosco and Checchi (2023) [18]	OM and VDO	Class V	In-office (Opalescence Boost 40%)	VITA Easyshade V	Grey	Baseline and 24 h post-bleaching	Distilled water at 37 °C	Excellent color match between restoration and teeth can be obtained, both before and after tooth bleaching.
Cubukcu, Gundogdu and Gul (2023) [23]	CL and OM	Cavities 3 × 5 width and 2 mm deeph (A3 shade)	In-office (Opalescence Boost 40%) and at-home (Opalescence Boost 16%)	Spectro ShadeTM Micro	White	Baseline, after staining process, 7, 14 and 28 days post-bleaching	Coffee for 5 days at room temperature	The color match between tooth and filling was better in the CL, and in-office bleaching had less impact on the color match.
Forabosco et al. (2023) [19]	OM, VDO, CL, ES-2, and ES	Class V	In-office (Opalescence Boost 40%)	VITA Easyshade V	*	Baseline, 24 h and 1 week post-bleaching	Distilled water at 37 °C	All single-shade RBCs tested showed the best color match with the surrounding tooth one week after bleaching.
Forabosco et al. (2024) [20]	OM, VDO, CL, ES-2 and ES	Class V	In-office (Opalescence Boost 40%)	VITA Easyshade V	Grey	Baseline, 24 h and 1 week post-bleaching	Distilled water at 37 °C	The four tested single-shade RBCs showed a good color match with the surrounding tooth after bleaching.
Razzaq, Refaat and Al-Badr (2024) [21]	OM	Class V	In-office (Power Whitening Gel with a 40%)	VITA Easyshade	White	Baseline, first application and second application)	Distilled water	OM can change its shade according to the shade of the natural tooth after external bleaching application.
Sugimura et al. (2024) [16]	AU, BU, OM, and TU	Cavities 3 × 2 in labial side (A3 shade)	In-office (Opalescence Boost 35%)	CMS-35F SQC	Black and white	First, second and third application	Previous black tea; Artificial saliva	A mismatch was observed in all tested resins after the second application, indicating that single-shade RBCs are unable to maintain a color match with enamel after office whitening.

* not mentioned. Legend = LR: luminosity from restoration; LE: luminosity from enamel; OM: Omnichroma; VE: Venus Diamond One; CL: Clearfil Majestic; ES-2: ES-2 Universal; ES: Essencia Universal; AU: A Uno Universal Basic; BU: Beautifil Unishade; TU: Transcend Universal Composite; VDO: Venus Diamond One.

**Table 4:** L*, ΔE00 and ΔEab values collected from studies.

Study	n	Type of Restoration	Time	L* Restoration	L* Enamel	ΔE00	ΔEab
Mohamed et al. (2020) [22]	10	-	Baseline	73 (4.7)	69 (4.0)	-	-
		-	24 h	72.7 (4.0)	70.5 (4.0)	-	-
		-	1 week	72.3 (4.5)	69.2 (4.5)	-	-
		-	2 weeks	72 (4.5)	68 (4.0)	-	-
		-	4 weeks	72 (4.3)	69 (4.2)	-	-
AlHabdan et al. (2022) [17]	40	Restoration	Pre-restoration	73.685 (9.059)	-	-	-
		-	After restoration	82.057 (4.768)	-	-	-
		-	Post-bleaching	87.330 (4.428)	-	3.529	-
		-	2 weeks post-bleaching	81.954 (6.060)	-	3.651	-
Cubukcu, Gundogdu and Gul (2023) [23]	80	Single-shade + in office Bleaching	Baseline	-	-	2.24 (0.65)	-
		-	After staining	-	-	3.72 (1.57)	-
		-	1 day after bleaching	-	-	14.49 (8.28)	-
		-	7 days after bleaching	-	-	3.45 (1.77)	-
		-	14 days after bleaching	-	-	3.89 (1.84)	-
		-	28 days after bleaching	-	-	3.17 (1.61)	-
		Control RBC + in office bleaching	Baseline	-	-	1.97 (0.89)	-
			After staining	-	-	2.77 (1.64)	-
			1 day after bleaching	-	-	8.88 (6.91)	-
			7 days after bleaching	-	-	2.88 (1.50)	-
			14 days after bleaching	-	-	2.82 (1.50)	-
			28 days after bleaching	-	-	3.03 (1.31)	-
		Single-shade + at home Bleaching	Baseline	-	-	2.42 (1.10)	-
			After staining	-	-	2.88 (1.66)	-
			1 day after bleaching	-	-	4.20 (2.33)	-
			7 days after bleaching	-	-	3.88 (1.63)	-
			14 days after bleaching	-	-	3.62 (1.69)	-
			28 days after bleaching	-	-	4.27 (2.17)	-
		Control RBC + at home Bleaching	Baseline	-	-	1.79 (1.09)	-
			After staining	-	-	2.81 (1.14)	-
			1 day after bleaching	-	-	5.05 (2.04)	-
			7 days after bleaching	-	-	4.01 (1.35)	-
			14 days after bleaching	-	-	3.51 (1.63)	-
			28 days after bleaching	-	-	5.76 (1.55)	-
Forabosco et al. (2023) [19]	80	OM	Baseline	-	-	7.6 (2.4)	-
			After 24 h	-	-	4.7 (1.9)	-
			After 1 week	-	-	4.3 (1.9)	-
		VE	Baseline	-	-	3.8 (1.5)	-
			After 24 h	-	-	2.7 (1.2)	-
			After 1 week	-	-	3.2 (1.9)	-
		ES	Baseline	-	-	4.2 (1.9)	-
			After 24 h	-	-	4.3 (2.1)	-
			After 1 week	-	-	2.9 (1.6)	-
		CL	Baseline	-	-	7.3 (2.0)	-
			After 24 h	-	-	3.9 (1.4)	-
			After 1 week	-	-	4.0 (1.1)	-
Forabosco et al. (2024) [20]	80	OM	Baseline	-	-	-	12.5 (4.7)
			After 24 h	-	-	-	7.7 (3.9)
			After 1 week	-	-	-	6.3 (2.6)
		VE	Baseline	-	-	-	6.9 (2.9)
			After 24 h	-	-	-	4.3 (2.0)
			After 1 week	-	-	-	5.0 (2.9)
		ES	Baseline	-	-	-	7.5 (3.9)
			After 24 h	-	-	-	6.6 (3.0)
			After 1 week	-	-	-	4.4 (2.4)
		CL	Baseline	-	-	-	12.6 (4.1)
			After 24 h	-	-	-	6.2 (2.4)
			After 1 week	-	-	-	6.1 (1.8)
Razzaq, Refaat and Al-Badr (2024) [21]	26	Single-shade	Baseline	85.49 (2.71)	-	-	-
			After 1 application	86.56 (3.10)	-	-	3.75 (2.23)
			After 2 applications	87.78 (2.86)	-	-	4.77 (2.44)
		Tooth	Baseline	79.63 (5.13)	-	-	-
			After 1 application	82.31 (5.03)	-	-	4.12 (2.31)
			After 2 applications	79.84 (3.25)	-	-	4.90 (2.29)
Sugimura et al. (2024) [16]	40	AU	Baseline	-	-	-	4.55 (1.56)
Resin Restoration/cervical area			1st application	-	-	-	7.01 (3.34)
			2nd application	-	-	-	8.99 (3.18)
			3rd application	-	-	-	9.94 (3.62)
		BU	Baseline	-	-	-	3.93 (1.48)
			1st application	-	-	-	3.96 (1.58)
			2nd application	-	-	-	4.62 (1.38)
			3rd application	-	-	-	4.80 (2.41)
		OC	Baseline	-	-	-	5.28 (2.28)
			1st application	-	-	-	5.82 (2.20)
			2nd application	-	-	-	5.07 (2.08)
			3rd application	-	-	-	5.82 (1.32)
		TU	Baseline	-	-	-	5.26 (1.40)
			1st application	-	-	-	5.26 (1.80)
			2nd application	-	-	-	5.64 (1.50)
			3rd application	-	-	-	5.32 (2.50)
Resin Restoration/incisal area		AU	Baseline	-	-	-	4.33 (1.36)
			1st application	-	-	-	6.92 (3.81)
			2nd application	-		-	7.90 (2.34)
			3rd application	-		-	7.93 (2.50)
		BU	Baseline	-		-	4.51 (1.38)
			1st application	-		-	5.21 (1.88)
			2nd application	-		-	5.30 (2.00)
			3rd application	-		-	5.25 (1.65)
		OC	Baseline	-		-	6.01 (2.13)
			1st application	-		-	6.53 (2.39)
			2nd application	-		-	6.08 (2.53)
			3rd application	-		-	6.82 (1.64)
		TU	Baseline	-		-	5.96 (2.10)
			1st application	-		-	5.70 (1.20)
			2nd application	-		-	5.33 (2.20)
			3rd application	-		-	5.25 (2.30)

Legend = LR: luminosity from restoration; LE: luminosity from enamel; OM: Omnichroma; VE: Venus Diamond One; CL: Clearfil Majestic ES-2 Universal; ES: Essencia Universal; AU: A Uno Universal Basic; BU: Beautifil Unishade; TU: Transcend Universal Composite.

**Table 5:** Risk of bias analysis.

Author/Year	D1: Bias in Planning and Allocation			D2: Bias in Sample/Specimen Preparation		D3: Bias in Outcome Assessment		D4: Bias in Data Treatment and Outcome Reporting	
	Control Group	Randomization	Sample Size Rationale and Reporting	Standardization of Samples and Materials	Identical Experimental Conditions Across Groups	Adequate and Standardized Testing Procedures and Outcomes	Blinding of the Test Operator	Statistical Analysis	Reporting Study Outcomes
Mohamed et al. (2020) [22]	S	I	N	I	S	S	NA	S	I
AlHabdan et al. (2022) [17]	S	N	N	I	S	I	NA	S	I
Forabosco and Checchi (2023) [18]	S	I	N	S	S	I	I	N	I
Cubukcu, Gundogdu and Gul (2023) [23]	S	I	N	S	S	S	I	S	S
Forabosco et al. (2023) [19]	S	I	N	S	S	I	S	S	S
Forabosco et al. (2024) [20]	S	I	N	S	S	S	N	S	S
Razzaq, Refaat and Al-Badr (2024) [21]	S	N	N	I	S	I	NA	S	S
Sugimura et al. (2024) [16]	S	S	N	I	S	S	N	S	S

Legend: S: sufficiently reported/adequate, I: insufficiently reported, N: not reported/not adequate, NA: not applicable.

## Data Availability

All the data are available within the manuscript.

## Supplementary Materials

The following supporting information can be downloaded at: https://www.mdpi.com/article/10.3390/jcm14093194/s1, Table S1: Preferred Reporting Items for Systematic Reviews and Meta-Analyses (PRISMA) guidelines. Ref. [33] is cited in the Supplementary Materials.
